# Compatibility of fosfomycin with different commercial peritoneal dialysis solutions

**DOI:** 10.1007/s10096-017-3051-3

**Published:** 2017-07-06

**Authors:** M. Kussmann, A. Baumann, S. Hauer, P. Pichler, M. Zeitlinger, M. Wiesholzer, H. Burgmann, W. Poeppl, G. Reznicek

**Affiliations:** 1 Department of Internal Medicine I, Division of Infectious Diseases and Tropical Medicine, Medical University Vienna, Vienna, Austria; 2 Department of Pharmacognosy, University of Vienna, Vienna, Austria; 3 Department of Internal Medicine I, University Hospital St. Poelten, Karl Landsteiner University of Health Sciences, St. Poelten, Austria; 4 Department of Clinical Pharmacology, Medical University Vienna, Vienna, Austria; 5 Military Medical Cluster East, Austrian Armed Forces, Vienna, Austria

## Abstract

For treatment of peritoneal dialysis-related peritonitis, intraperitoneal administration of antibiotics remains the preferable route. For home-based therapy, patients are commonly supplied with peritoneal dialysis fluids already containing antimicrobial agents. The present study set out to investigate the compatibility of fosfomycin with different peritoneal dialysis fluids, namely, Extraneal^®^, Nutrineal^®^, Physioneal^®^ 1.36% and Physioneal^®^ 2.27%, under varying storage conditions. The peritoneal dialysis fluid bags including 4 g fosfomycin were stored over 14 days at refrigeration temperature (6°C) and room temperature (25°C) and over 24 h at body temperature (37°C). Drug concentrations over time were determined by using high-performance liquid chromatography coupled to a mass spectrometer. In addition, drug activity was assessed by a disk diffusion method, diluent stability by visual inspection and drug adsorption by comparison of the measured and calculated concentrations. Blank peritoneal dialysis fluids and deionized water were used as comparator solutions. Fosfomycin was stable in all peritoneal dialysis fluids and at each storage condition investigated over the whole study period. The remaining drug concentrations ranged between 94% and 104% of the respective initial concentrations. No significant drug adsorption was observed for any peritoneal dialysis fluid at any storage condition. No relevant reduction of antimicrobial activity was observed. Fosfomycin is compatible with Extraneal^®^, Nutrineal^®^ and Physioneal^®^ for up to two weeks at refrigeration or room temperature and may be used for home-based therapy. No dose adjustment is needed due to adsorption or degradation.

## Introduction

Peritoneal dialysis-related peritonitis (PDRP) remains a common and serious complication of peritoneal dialysis (PD) which may result in peritoneal membrane failure and conversion to long-term hemodialysis [1]. The most common pathogens isolated from patients with PDRP are mainly gram positive cocci like Staphylococci or Streptococci, but also gram negative bacteria like *Escherichia coli* or *Pseudomonas aeruginosa* are frequently detected [2–4]. Therefore, empirical antimicrobial regimens for treatment of PDRP should cover both gram positive and gram negative bacteria in consideration of center-specific resistance patterns [1]. According to the ISPD recommendations, the intraperitoneal (IP) administration of antibiotics admixed to peritoneal dialysis fluids (PDF) should be the preferred route allowing higher concentrations at the target site, avoiding venipuncture and reducing gastrointestinal toxicities [1, 5]. For home-based therapy of PDRP, patients are commonly supplied with PDFs already containing antimicrobial agents. These PDF bags are then warmed up to body temperature by a heating plate at home directly before administration [1, 6]. Fosfomycin is a bactericidal agent with longstanding clinical use in a wide range of patient populations. It is active against a broad spectrum of gram positive and gram negative bacteria including difficult-to-treat pathogens like MRSA, *P. aeruginosa* or extended-spectrum-β-lactamase-producing *Enterobacteriaceae*. It is well tolerated and displays a favorable safety profile [7–13]. Thus, fosfomycin could be a valuable alternative for the treatment of PDRP. For IP administration, however, data on drug stability and compatibility with commercially available PDFs are prerequisite to ensure sufficient peritoneal drug exposure. Thus, the present in vitro study set out to investigate the stability and compatibility of fosfomycin with four different commercially available PDFs under varying storage conditions.

## Materials and methods

The compatibility of fosfomycin with four different commercially available PDFs (Extraneal^®^ 2000 ml, 75 g/l icodextrin; Nutrineal^®^ 2500 ml, 1.1% amino acids; Physioneal^®^ 40 1.36% glucose 2000 ml [chamber A 725 ml, chamber B 1275 ml]; Physioneal^®^ 40 2.27% glucose 2000 ml [chamber A 725 ml, chamber B 1275 ml]; all Baxter Healthcare Corp., Deerfield, IL, USA) was investigated at three different temperatures: over 14 days at refrigeration temperature (6 °C) and room temperature (25 °C) and over 24 h at body temperature (37 °C). Fosfomycin (Fosmicin 4 g, Meiji Seika Pharma Co. Ltd.) was obtained in the form of dry powder and diluted according to the manufacturer’s instructions. The solutions containing 4 g of fosfomycin were injected into the dialysis bags. For the two-chamber bag system of Physioneal^®^, fosfomycin was administered into the low pH chamber A (containing 725 ml) which remained separated when compatibility was evaluated over 14 days at refrigeration or room temperature. For the evaluation at body temperature, the two chambers of Physioneal^®^ were mixed immediately after the injection of fosfomycin before the PDF bags were placed on a heating plate (PD Bag Warmer, Baxter) for 60 min. Thus, the calculated fosfomycin concentrations were 1980 mg/L in Extraneal^®^, 1587 mg/L in Nutrineal^®^ , and for the two-chambered Physioneal^®^ bags 5369 mg/L for the unmixed solutions at 6 °C and 25 °C and 1980 mg/L for the mixed solutions at 37 °C. Altogether, 48 PDF bags (12 bags per PDF) were used in the present study. For compatibility evaluation of fosfomycin, three bags per PDF were stored at each temperature investigated and additionally one bag without study drug was run as control to evaluate the diluent stability at each storage condition as determined by visual inspection. All PDF bags were stored light-protected in temperature-controlled rooms and samples of 30 ml were taken in duplicate at the following time points: 0 (directly after addition of fosfomycin), 6, 24, 48, 72, 168, 336 h for storage at refrigeration and room temperature (25 °C) and 0, 1, 2, 4, 12, 24 h for storage at body temperature (37 °C). The PDF bags were thoroughly shaken before each sample collection and the samples were stored at −80 °C until analysis. In addition, the compatibility of equal fosfomycin concentrations was evaluated in aqueous solution in a glass container. The concentrations of fosfomycin were determined by using high-performance liquid chromatography coupled to a mass spectrometer (LC-MS). Drug stability was defined as a drug decomposition of less than 10% and drug adsorption (drug-container interaction) was assessed by comparison of the calculated target concentration with the initial measured fosfomycin concentration obtained directly after administration [4]. In addition, to verify antimicrobial activity of fosfomycin after exposure to different storage conditions and periods, a disk diffusion inhibition assay with *Escherichia coli* (ATCC 25922) was performed in duplicates at each time point of sample collection. Therefore, after an incubation of 24 h at 36 °C on Columbia Agar plates (5% sheep blood) the inhibitory zone diameters were measured and compared to initial inhibitory diameters obtained. For quality assurance of the diffusion disk inhibition assay, fosfomycin diluted in aqueous solution as well as all PDFs without fosfomycin were run as control. For evaluation of the diluent stability, PDF bags were visually inspected for color change, particulate matter or haze and the pH was measured at each time point of sample collection. For sterility testing, 500 μL of each sample were filled into 10 mL tubes containing thioglycollate to detect contamination with anaerobe and tryptone soya broth (both from Oxoid Deutschland GmbH; Wesel, Germany) for contamination with aerobe organism. After 10 days of incubation at 37 °C the tubes were checked visually for clouding, particulate matter and turbidity.

### Sample analysis by LC-MS

For the chemical analysis of fosfomycin stability, the samples (5 μl) were analyzed using liquid chromatography/mass spectrometry (LC-MS/MS) on an Ultimate 3000 RSLC-series system (Thermo Fisher Scientific, Sunnyvale, California, USA) coupled to a triple quadrupol mass spectrometer (AB Sciex Instruments API 4000, Concord, Ontario, Canada) equipped with an orthogonal ESI source operated in negative mode. LC separation was performed on an Acclaim RSLC 120 C18 column (3 μm, 100 × 2.1 mm I.D., Thermo Fisher Scientific), preceded by an Acclaim 120 C18 guard cartridge (5 μm, 10 × 2 mm I.D., Thermo Fisher Scientific), at a flow rate of 0.4 mL/min and a column temperature of 25 °C. The mobile phase consisted of 1% aqueous formic acid (mobile phase A), and acetonitrile containing 1% formic acid (mobile phase B). The analysis was carried out in isocratic mode with 70% mobile phase B and 30% mobile phase A with a total analysis time of 3 min. Fosfomycin eluted at 1.06 min. Selective and sensitive detection and quantification was carried out using MS/MS fragmentation of fosfomycin giving a quasimolecular ion at m/z 137,0 [M-H]^−^. MRM m/z 137,0/78,8 (quantifier) as well as MRM m/z 137,0/63,0 (qualifier) were used for calibration curves with external standard fosfomycin (injection volume 5 μl) to give a linear concentration range from 0.1 mg/mL to 10 mg/mL (correlation coefficient 0.9995). The triple quadrupol mass spectrometer operated with the following parameters: ESI neg., IS -4500, EP -10, CUR 10, GS1 40, GS2 40, TEM 500 °C, CAD 4, CEM 2200, DF 200. MRM 137,0/78,8: DP -30, CE -24, CXP -3, dwell 150 ms. MRM 137,0/63,0: DP -30, CE -22, CXP -7, dwell 150 ms. After collection the samples were analyzed immediately. Preliminary investigations showed that the storage of the samples in the autosampler at 10 °C till analysis did not affect the fosfomycin concentrations at all (data not shown). Each sample series was analyzed within one day, the single samples were analyzed in triplicate and the mean value was calculated. Each sample series was interspersed with several quality control (QC) samples of known concentrations to ensure the validity of the results. Fosfomycin gave an isolated peak in the total ino current (TIC) with nice peak shape (symmetry factor 1.0 to 1.15) and no degradation products were detected in any of the sample solutions. System suitability test of the standard solution gave 0.29% RSD (peak area, *n* = 6). To test the specificity of the method, the single PDFs were injected and no peak could be detected. No carry over could be seen after consecutive injection (10 times) of the standard solution. The precision of the method (fosfomycin solution in PDF) gave 0.53% RSD (peak area, *n* = 6). The concentrations given in Table 1 are mean values from three PD bags (each two samples were drawn and analyzed in triplicate), in total 18 analyses per time point and temperature.

**Table 1:** Mean concentrations of fosfomycin (mg/mL) in different peritoneal dialysis fluids, at various temperatures and time points

Temperature	Time (hours)	Extraneal			Nutrineal			Physioneal 1.36%			Physioneal 2.27%		
		Conc (mg/mL)	% RSD	% IC	Conc (mg/mL)	% RSD	% IC	Conc (mg/mL)	% RSD	% IC	Conc (mg/mL)	% RSD	% IC
**6 °C**		***CTC: 1.980***		*100.00*	***CTC: 1.587***		*100.00*	***CTC: 5.369***		*100.00*	***CTC: 5.369***		*100.00*
	0	1.980	3.02	**100.00**	1.588	1.94	**100.06**	5.275	4.93	**98.25**	5.332	2.46	**99.31**
	6	1.975	3.58	**99.75**	1.570	2.18	**98.93**	5.077	3.67	**94.56**	5.300	2.42	**98.71**
	24	1.972	1.25	**99.60**	1.549	1.77	**97.61**	5.330	1.54	**99.27**	5.358	1.91	**99.80**
	48	1.975	1.58	**99.75**	1.511	4.12	**95.21**	5.256	2.82	**97.90**	5.129	1.41	**95.53**
	72	1.960	1.80	**98.99**	1.533	5.26	**96.60**	5.384	2.63	**100.28**	5.029	5.69	**93.67**
	168	1.932	3.08	**97.58**	1.632	2.65	**102.84**	5.473	3.19	**101.94**	5.241	1.07	**97.61**
	336	1.863	5.07	**94.09**	1.611	6.79	**101.51**	5.115	5.21	**95.27**	5.236	2.98	**97.53**
**25 °C**		***CTC: 1.980***		*100.00*	***CTC: 1.587***		*100.00*	***CTC: 5.369***		*100.00*	***CTC: 5.369***		*100.00*
	0	1.980	2.34	**100.00**	1.587	0.258	**100.00**	5.369	2.35	**100.00**	5.369	2.66	**100.00**
	6	1.998	2.91	**100.91**	1.491	1.31	**93.95**	4.940	3.03	**92.01**	5.226	1.35	**97.34**
	24	2.013	2.13	**101.67**	1.507	3.73	**94.96**	5.341	2.58	**99.48**	5.192	1.97	**96.70**
	48	2.000	2.49	**101.01**	1.468	3.68	**92.50**	5.429	2.80	**101.12**	5.172	3.01	**96.34**
	72	1.981	1.40	**100.05**	1.491	4.47	**93.95**	5.584	2.27	**104.00**	5.269	2.86	**98.14**
	168	1.984	2.43	**100.20**	1.539	1.22	**96.98**	5.479	2.09	**102.05**	5.353	1.30	**99.70**
	336	1.984	2.07	**100.20**	1.508	3.01	**95.02**	5.357	2.85	**99.78**	5.322	3.18	**99.12**
**37 °C**		***CTC: 1.980***		*100.00*	***CTC: 1.587***		*100.00*	***CTC: 1.980***		*100.00*	***CTC: 1.980***		*100.00*
	0	1.980	3.10	**100.00**	1.587	2.47	**100.00**	1.980	4.12	**100.00**	1.980	2.78	**100.00**
	1	1.783	4.75	**90.05**	1.466	3.76	**92.38**	1.895	3.52	**95.71**	1.994	1.40	**100.71**
	2	1.809	4.13	**91.36**	1.548	1.32	**97.54**	2.041	3.53	**103.08**	1.874	1.78	**94.65**
	4	1.849	1.59	**93.38**	1.542	3.66	**97.16**	2.014	2.76	**101.72**	1.872	2.47	**94.55**
	12	1.916	4.01	**96.77**	1.553	1.43	**97.86**	2.009	4.43	**101.47**	1.879	3.98	**94.90**
	24	1.858	1.60	**93.84**	1.534	3.28	**96.66**	1.970	1.84	**99.49**	1.925	4.60	**97.22**

*Conc* concentration, *%RSD* percentage relative standard deviation, *% IC* percentage of the initial concentration, *CTC* calculated target concentration

## Results

The mean concentrations and inhibitory zone diameters over time of fosfomycin in four different commercially available PDFs are outlined in Tables 1 and 2, respectively. Defined as a decomposition of ≤10% compared to the initial concentration, fosfomycin was stable in all PDFs and at each storage condition investigated over the whole study period. The remaining drug concentrations in Extraneal^®^, Nutrineal^®^, Physioneal^®^ 1.36% and Physioneal^®^ 2.27%, after 14 days at refrigeration and room temperature as well as after 24 h at body temperature, ranged between 94% and 104% of the respective initial concentrations (Table 1). With a maximum difference of 1.75% between the calculated target concentration and the measured initial concentration (in Physioneal^®^ 1.36%), no relevant initial adsorption could be observed in any PDF at any storage condition investigated. The *E. coli* diffusion disk inhibition assay revealed no relevant reduction of antimicrobial activity of fosfomycin in any PDF over the whole study period at any temperature condition investigated (Table 2). The PDFs without study drug showed no antimicrobial activity and the inhibitory zone diameters of fosfomycin in aqueous solutions were equal to results from PDF solutions. No abnormalities were observed by visual inspection or pH measurement over the whole study period neither in drug containing nor control PDF bags. No microbial contamination could be detected by sterility testing in any sample. In parallel the stability of fosfomycin in water was evaluated. Respective aqueous solutions were prepared with double distilled water in glass containers and stored at the same conditions (temperature, time) as the PD solutions. At 6 °C, 25° as well as 37 °C no decomposition of fosfomcyin could be found within the complete observation periods.

**Table 2:** Inhibitory zone diameters of the *Escherichia coli* inhibiton assay in % of the initial zone diameters (% IZD) in different peritoneal dialysis fluids at various temperatures and time points

Temp.	Time (hours)	Extraneal		Nutrineal		Physioneal 1.36%		Physioneal 2.27%	
		%IZD	%RSD	%IZD	%RSD	%IZD	%RSD	%IZD	%RSD
**6 °C**	0	100.0	1.2	100.0	0.0	100.0	1.1	100.0	0.0
	6	99.2	0.0	102.6	1.2	100.8	0.0	99.3	1.0
	24	100.0	1.2	101.7	0.0	102.3	0.0	100.0	0.0
	48	98.3	1.2	102.6	1.2	102.3	0.0	99.3	1.0
	72	100.8	0.0	103.4	0.0	103.1	1.1	100.0	0.0
	168	99.2	2.4	102.6	1.2	101.5	1.1	100.7	1.0
	336	97.5	0.0	103.4	0.0	103.1	1.1	100.7	1.0
**25 °C**	0	100.0	0.0	100.0	1.2	100.0	0.0	100.0	1.1
	6	101.7	0.0	99.2	0.0	97.8	1.0	100.8	0.0
	24	100.8	1.2	98.3	1.2	99.3	1.0	100.8	2.2
	48	102.5	1.2	99.2	0.0	98.6	0.0	103.8	2.2
	72	103.3	0.0	98.3	1.2	98.6	0.0	103.1	1.1
	168	101.7	0.0	100.0	1.2	97.8	1.0	102.3	2.2
	336	100.0	0.0	100.0	1.2	98.6	0.0	102.3	2.2
**37 °C**	0	100.0	0.0	100.0	0.0	100.0	0.0	100.0	0.0
	1	101.7	0.0	100.8	1.2	99.2	1.2	100.0	2.3
	2	101.7	0.0	101.7	0.0	100.8	1.2	100.0	0.0
	4	100.8	1.2	104.2	1.2	101.7	2.4	100.0	0.0
	12	101.7	0.0	105.1	0.0	101.7	0.0	101.6	2.3
	24	101.7	0.0	103.4	0.0	101.7	2.4	100.8	1.1

*%RSD* percentage relative standard deviation

## Discussion

Several studies highlighted that fosfomycin could serve as an important alternative antimicrobial agent in the treatment of various infections in particular when caused by difficult-to-treat pathogens [7–10]. Before the clinical use of intraperitoneal fosfomycin, however, it is important to ensure compatibility of the antibiotic agent with PDFs in which the drug is administered. Quentin et al. [14] investigated the stability of fosfomycin in the 1.36% glucose containing PDF Dianeal^®^ . Drug concentrations were determined by the use of a microbiological assay and fosfomycin was found to be stable (drug decomposition less than 10%) at room temperature over 24 h . However, in that study no other PDFs, storage conditions, longer time periods, or other important aspects of drug-diluent compatibility like drug adsorption or diluent stability were investigated. Further, according to current recommendations by De Vin et al. [6], compatibility data obtained in a specific PDF should not be extrapolated to other solutions due to significant differences in the composition. In the present study the compatibility of fosfomycin with four different commercially available PDFs was investigated. Fosfomycin was shown to be stable over a period of two weeks at refrigeration and room temperature. Likewise, diluent stability was shown not to be affected by the addition of fosfomycin. Thus, when used for home-based antimicrobial therapy of PDRP, patients can be supplied with PDFs containing fosfomycin. Compared with drug degradation, drug adsorption is a faster process and can already be seen directly after the antibiotic administration [6]. These potential drug-container interactions were examined by comparison of the calculated drug concentrations with the measured initial concentrations and were found to be negligible. In clinical routine, PDF bags are usually warmed up to body temperature by a heating plate before application. In the present study it was demonstrated that this heating process had no impact on fosfomycin stability, diluent stability, or drug adsorption. Thus, patients can be supplied with PDFs containing fosfomycin and no compensatory increase of the dosage is needed for home based therapy of PDRP. Noteworthy, recent studies have shown that the in vitro activity of antibiotics might be significantly reduced in commercially available PDFs [15–19]. In particular for time dependent antimicrobials, including ampicillin, cefepime, ertapenem, meropenem, linezolid and glycopeptide antibiotics, a strongly reduced in vitro activity was observed. Fosfomycin, however, is a broad spectrum antimicrobial agent which displays time-, but also concentration-dependent activity [20–24]. In the present study, aside the chemical stability over time measured with (LC-MS/MS) also the antimicrobial activity of fosfomycin was found to be preserved over the whole study period as shown by the results of the disk diffusion assay. Of interest, there is some literature that fosfomycin was already used IP in patients with PDRP and the pharmacokinetics of IP fosfomycin were investigated in patients with CAPD and APD [25–29]. A study by Tobudic et al. investigated the pharmacokinetic properties of IP and IV fosfomycin in eight non-infected patients undergoing automated PD (APD) [29]. After IP administration of 4 g fosfomycin T > MIC values of 97.61% (±1.4), 89.58% (±5.5) and 49.1% (±28.1) in serum as well as 100.0% (±0), 73.21% (±12.3) and 65.16% (±16.5) in dialysate could be observed for MICs of 4 mg/L, 64 mg/L and 128 mg/L, respectively. In contrast, after IV administration, T > MIC values were 100.0% (±0), 98.95% (±2.9) and 79.69% (±21.8) in serum and 50.3% (±49.04), 30.21% (±43.46) and 17.97% (±28.85) in dialysate for MICs of 4 mg/L, 64 mg/L and 128 mg/L, respectively. The ratio of IP to systemic exposure was 1.12 whereas the ratio of IV to peritoneal exposure was only 0.22. This indicates excellent absorption of IP fosfomycin and underlines its potential usefulness for IP treatment of PDRP as well as systemic infections. In contrast, the poor penetration of IV fosfomycin into the abdominal cavity limits its IV use for treatment of PDRP. The emergence of drug resistant bacteria might foster the reintroduction of this antimicrobial agent in the management of patients with PDRP. In conclusion, fosfomycin is compatible with Extraneal^®^, Nutrineal^®^ and Physioneal^®^ and may be used for inpatient and also for home-based therapy in patients with PDRP. Further studies investigating the clinical efficacy of IP fosfomycin are warranted.
